# Back Pain and Heart Failure in Older Adults: Findings From the Health ABC Study

**DOI:** 10.1093/geroni/igaa057.656

**Published:** 2020-12-16

**Authors:** Jie Chen, Yiming Zhang, Eleanor Simonsick, Angela Starkweather, Ming-Hui Chen, Paula McCauley, Kendra Maas, Xiaomei Cong

**Affiliations:** 1 University of Connecticut, Storrs, Connecticut, United States; 2 National Institute on Aging, Bethesda, Maryland, United States; 3 UConn Health, Farmington, Connecticut, United States

## Abstract

Both back pain and heart failure (HF) have negative influence on all aspects of life. Little is known about the impact of back pain on older adults with HF. We include 1295 older adults who had data collected in the 11th year (2007-2008) of the Health, Aging and Body Composition (Health ABC) study to evaluate the effect of back pain on health status among older adults with and without HF. The participants aged 79-91, 54.8% were female and 34.8% were African American. Among 94 participants with HF, 63 (67.0%) had back pain; among 1201 participants without HF, 649 (54.0%) had back pain. Females reporting back pain had 4.76 (95% CI: 1.83, 12.37) times the odds of having HF compared to those without back pain. Male with back pain, compared to those without back pain, had 1.14 times (95% CI: 0.65, 2.02) the odds of having HF. Depressive symptoms were measured by the Center for Epidemiological Studies-Depression (CES-D) scale. Performance and functions were measured by the Established Populations for Epidemiologic Studies in the Elderly (EPESE) performance score, the Health ABC performance battery score and self-reported difficulty with functional tasks. These symptom and performance measures were significantly associated with both back pain and HF, but not the interaction terms of back pain and HF after adjusting demographic variables including gender, race, smoking status and BMI category. The high incidence and negative impact of back pain highlighted the needs of developing strategies in pain management among older adults with and without HF.

